# Cisplatin-resistant cells in malignant pleural mesothelioma cell lines show ALDH^high^CD44^+^ phenotype and sphere-forming capacity

**DOI:** 10.1186/1471-2407-14-304

**Published:** 2014-04-30

**Authors:** Lourdes Cortes-Dericks, Laurene Froment, Ruben Boesch, Ralph Alexander Schmid, Golnaz Karoubi

**Affiliations:** 1 Department of Clinical Research, Division of General Thoracic Surgery, University Hospital Berne, Berne, Switzerland

**Keywords:** Cancer stem cells, Malignant pleural mesothelioma, Chemoresistance, ALDH, CD44

## Abstract

**Background:**

Conventional chemotherapy in malignant pleural mesothelioma (MPM) has minimal impact on patient survival due to the supposed chemoresistance of cancer stem cells (CSCs). We sought to identify a sub-population of chemoresistant cells by using putative CSC markers, aldehyde dehydrogenase (ALDH) and CD44 in three MPM cell lines; H28, H2052 and Meso4.

**Methods:**

The Aldefluor assay was used to measure ALDH activity and sort ALDH^high^ and ALDH^low^ cells. Drug-resistance was evaluated by cell viability, anchorage-independent sphere formation, flow-cytometry and qRT-PCR analyses.

**Results:**

The ALDH^high^ - and ALDH^low^ -sorted fractions were able to demonstrate phenotypic heterogeneity and generate spheres, the latter being less efficient, and both showed an association with CD44. Cis- diamminedichloroplatinum (II) (cisplatin) treatment failed to reduce ALDH activity and conferred only a short-term inhibition of sphere generation in both ALDH^high^ and ALDH^low^ fractions of the three MPM cell lines. Induction of drug sensitivity by an ALDH inhibitor, diethylaminobenzaldehyde (DEAB) resulted in significant reductions in cell viability but not a complete elimination of the sphere-forming cells, suggestive of the presence of a drug-resistant subpopulation. At the transcript level, the cisplatin + DEAB-resistant cells showed upregulated mRNA expression levels for ALDH1A2, ALDH1A3 isozymes and CD44 indicating the involvement of these markers in conferring chemoresistance in both ALDH^high^ and ALDH^low^ fractions of the three MPM cell lines.

**Conclusions:**

Our study shows that ALDH^high^ CD44^+^ cells are implicated in conveying tolerance to cisplatin in the three MPM cell lines. The combined use of CD44 and ALDH widens the window for identification and targeting of a drug-resistant population which may improve the current treatment modalities in mesothelioma.

## Background

Malignant pleural mesothelioma is an aggressive tumour with a very poor prognosis [1]. The combination of pemetrexed and cisplatin is considered the front-line regimen for this disease, yielding a response rate of 41% and a median survival of 12.1 months [2]. Despite continuous efforts to implement new therapeutic modalities, none of these, have prolonged patient survival primarily due to chemoresistance [1]. It has been hypothesized that tumour relapse may be associated with the drug resistance of cancer stem cells; a rare cell population with the exclusive ability to self- renew and maintain a tumour [3]. Hence, the identification and complete elimination of these cells presents an ultimate goal in MPM therapy. Current studies have identified ALDH and CD44 as putative CSC markers which exhibit high chemoresistive properties in solid cancers; thus, rendering them as potential indicators of drug tolerance in MPM.

Aldehyde dehydrogenase enzymes are a family of intracellular enzymes that are involved in cellular proliferation, differentiation, detoxification, and drug resistance through the oxidation of cellular aldehydes [4,5]. Certain ALDH isozymes are upregulated in tumour cells resulting in decreased cellular sensitivity to cyclophosphamides and some oxazaphosphorines leading to decreased chemotherapeutic effect. In leukemia and lung cancer cell lines overexpression of ALDH1A2a and ALDH2 increased cell proliferation and resistance to 4-hydroperoxicyclophosphamide and doxorubicin indicating a role for the ALDH isozymes in drug resistance [6]. The modulation of ALDH activity has been a central subject of research to improve the efficacy of conventional chemotherapeutic drugs. Studies have shown that in small cell lung cancer, lung adenocarcinoma, leukemia and lung cancer cell lines the ALDH-dependent chemoresistance can be inhibited by DEAB or siRNA that conferred sensitivity to drug treatments [7-9].

CD44, a transmembrane receptor for hyaluronan is associated with aggressive tumour growth, metastasis and resistance to therapy [10,11]. In combination with other surface markers (e.g., ALDH and CD24), CD44 can discriminate between various cancer subsets. In solid cancers the ALDH^high^/CD44^+^ subpopulation has been shown to possess stem cell-like properties that convey radio- and chemoresistance [12-14]. ALDH^+^/CD44^+^ subpopulation can be sensitized by selective inhibition of ALDH activity using DEAB or all-*trans* retinoic acid, ATRA in breast cancer [14]. As a single marker, CD44 is currently considered as a putative CSC indicator in human carcinomas including cancer of the lung. In NSCLC cell lines, sorted CD44^+^ cells that bear stem cell-like properties conferred more resistance to cisplatin exhibiting lower apoptotic levels compared with CD44^-^ cells [15].

Despite the current evidence linking ALDH and CD44 to drug resistance in solid tumours, the variability in the different studies still warrants further investigation to delineate the present roles of these potential CSC markers. Here, we sought to investigate whether ALDH can select for a drug-resistant subpopulation in three MPM cell lines. We also assessed whether the ALDH^high^ cells were associated with CD44, thus broadening the spectrum for identification of a drug-tolerant subpopulation in MPM. The specific selection of a chemoresistant subpopulation using ALDH and CD44 may serve as a potential therapeutic target that may be employed as adjuvant therapy to the current standard treatment modalities in MPM.

## Methods

### Cell culture

The H28 and H2052 mesothelioma cell lines (LCD Promochem, France) were maintained in RPMI 1640 (PAA, Austria) containing 10% fetal bovine serum, FBS (PAA, Austria) and 1% penicillin/streptomycin solution (Invitrogen, Switzerland). ACC-Meso-4 cell line was purchased from Riken Cell Bank, Resource No: RBRC-RCB2293 (Ibaraki, Japan) and cultured using the above-mentioned culture medium. Cells were cultured at 37°C, 95% humidity and 5% C0_2_. The general information issued by the providers of the three MPM cell lines does not have data on drug resistance to cisplatin.

### Sphere formation

Single-cell preparations of parental and ALDH-sorted MPM cell lines were resuspended in an appropriate amount of sphere-forming medium (RPMI1640 supplemented with 20 ng/ml EGF and bFGF, [Invitrogen, Switzerland]; 4 μg/ml insulin, [Sigma, Germany]; 1 ml B27, [Invitrogen, Switzerland] and 1% penicillin/streptomycin solution). For all cell lines, 5 x 10^3^ cells/ml/well were seeded onto a 24-well ultra-low adherent plate (Costar, USA). Cells were incubated at 37°C, 95% humidity and 5% C0_2_ for 7–14 days. The documentation of images and evaluation of sphere-forming efficiency were performed on day 7. Sphere-forming efficiency (%) was determined by dividing the number of spheres formed by the original number of seeded cells. The quotient was then multiplied by 100 [16]. Images were taken with Leica DMI 4000B at 5x magnification.

### Drug treatment

Drug resistance to cisplatin of mesothelioma cells were assessed by exposure to the IC_50_ values obtained for the non-sorted and ALDH-sorted cells for each of the three MPM cell lines. For the determination of IC_50_, a dilution series of 2-fold increments of cisplatin (0–256 μM Cisplatin, CDDP, Bristol Myers Squibb, Switzerland) were prepared in RPMI 1640 supplemented with 10% FBS and 1% penicillin/streptomycin. Cells at a density of 5 x 10^3^cells/100 μl/well in 96-well plates were incubated in media with or without the addition of cisplatin. Following a 48- and 72-hr incubation periods, culture media was aspirated, then replenished with XTT cell proliferation assay (Roche Chemicals, Switzerland) reagents. After a 30-min incubation at 37°C, formazan production was measured spectrophotometrically at 450 nm. Three independent experiments in triplicate were performed.

For cisplatin treatment, cells were cultured at 5 x 10^4^ cells/well in a 6-well plate (in three replicates) 48 hours prior to the addition of the previously determined IC_50_ of cisplatin for each cell line in RPMI 1640 containing 10% FBS and 1% penicillin/streptomycin solution. Following the 48- and 72-h hour treatments at 37°C, cells were washed with PBS and harvested to perform the following: mRNA isolation, sphere formation assay and cell viability. Pre-treatment of cells with 100 μM of ALDH inhibitor, DEAB (Sigma, Germany) was done for 48 h prior to cisplatin treatment [6,14].

### Aldefluor assay and flow cytometry

The Aldefluor kit (Stem Cell Technologies, Canada) was used to identify the cells expressing ALDH activity. Cells (0.5 – 1.0 x 10^6^) were incubated in assay buffer containing ALDH substrate BODIPY-aminoacetaldehyde (BAAA). As negative control, an aliquot of the ALDH substrated-treated cells were immediately quenched with specific ALDH inhibitor, DEAB. Both tubes were incubated for 45 min at 37°C. After incubation, cells were centrifuged and the pellets were resuspended with 500 μl of assay buffer prior to data acquisition using the green fluorescence channel of the LSR II flow cytometer (Becton Dickinson). This flow cytometry-based assay is termed as FACS analysis in this manuscript. DEAB-treated cells served as control to set the ALDH^high^ regions. The same staining procedure was applied before sorting the cells with FACS Aria using FACS Diva software (Becton Dickinson). Cell purity was determined at each independent sorting. After sorting of the ALDH^high^ and ALDH^low^ fractions, these were immediately used in all experiments. ALDH^high^CD44^+^ phenotype was assessed by immediately re-staining the freshly-sorted ALDH^high^ and ALDH^low^ cells with mouse anti-human CD44 APC-H7 (clone G44-26, BD Pharmingen, USA) and appropriate isotype control (mouse IgG_2b_K) for 30 min on ice in the dark. After washing with FACS buffer, data acquisition followed immediately. Propidium iodide (PI) staining was used to exclude the non-viable cells. FACS data were analysed with Flowjo software 7.2.5 (Treestar, Oregon, USA).

### RNA extraction and real-time quantitative PCR

Cell cultures were collected in an appropriate amount of RNAprotect™ cell reagent (Qiagen, Germany) followed by total RNA extraction using RNeasy Kit ( Qiagen) according to manufacturer’s instructions. Complementary DNA (cDNA) was synthesized by using the High capacity cDNA reverse transcription kit (Applied Biosystems, Rotkreuz, Switzerland) following the manufacturer’s instructions. The mRNA levels of the housekeeping gene *β2-microglobulin, β2M,* and the target genes ALDH1A1, ALDH1A2, ALDH1A3, ALDH2 and CD44 were quantified with the commercially available TaqMan “Assay on Demand” primer/probes (*β2M* – *Hs 99999903*_*m1; ALDH1A1 –Hs 00167445_m1; ALDH1A2- Hs 0018025_m1; ALDH1A3 –Hs 00167476_m1; ALDH2 – Hs 010007998_ml; CD44 –Hs01075861_m1*) (Applied Biosystems; Rotkreuz, Switzerland). Twenty nanograms of resulting cDNAs were subjected to quantitative RT-PCR, in a 10 μl final reaction volume and analyzed in triplicates. Gene expression was detected using ABI 7900 sequence detection system. The gene expression level of each target gene was normalized by the endogeneous gene, *β2M* and compared among cells by the ΔΔCT method. Baseline and threshold for Ct calculation were set automatically with ABI Prism SDS 2.1 software.

### Statistical analysis

Statistical analyses were performed using Graph Pad Prism 5.0 software© (San Diego, Ca). Two-tailed Student’s *t*-test was used to compare 2 groups. One- or two-way ANOVA with Bonferroni post hoc test as appropriate was performed to compare the values of >2 groups. The statistical significance was set at *p* <0.05.

## Results

### MPM cell lines exhibit sphere-forming cells and show different degrees of ALDH activity

**Figure 1:**
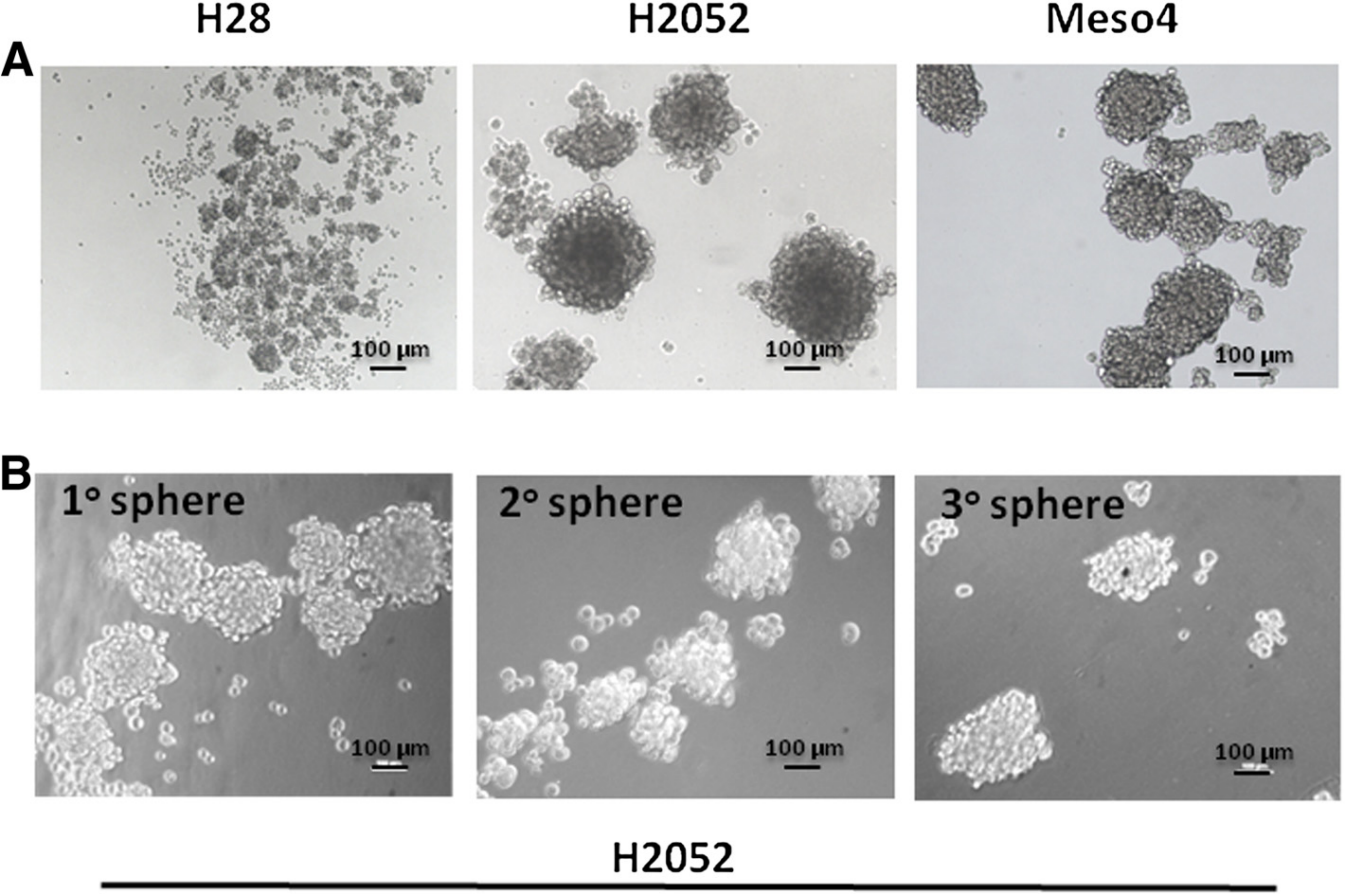
**MPM cell lines contain sphere-forming cell population. (A)** H28, H2052 and Meso4 grown under anchorage-independent culture conditions at 5000 cells/ml/well generated spheres of varying sizes and showed different sphere-forming efficiencies. **(B)** Representative images of sphere formation taken on day 7 on three consecutive generations.

**Figure 2:**
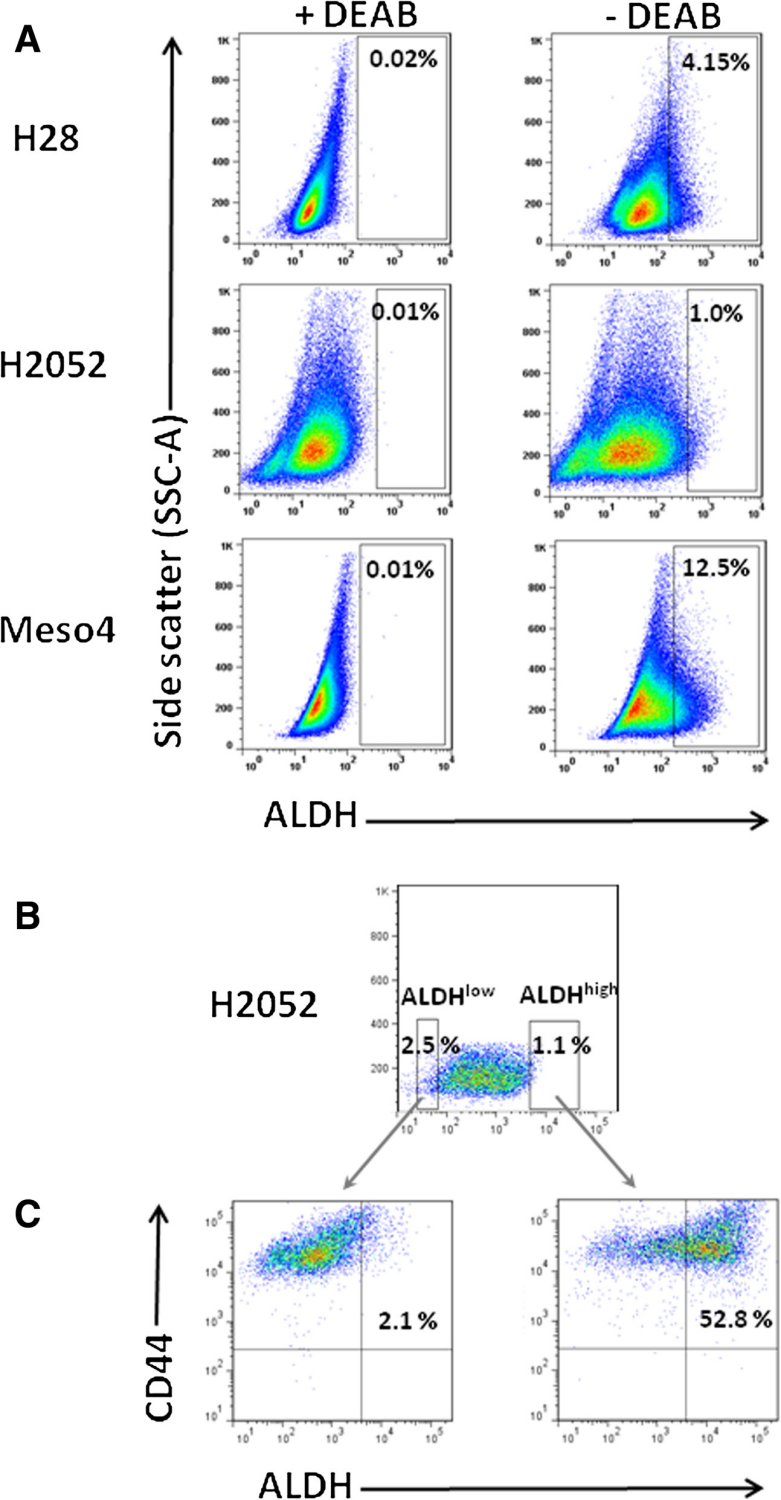
**Detection of ALDH activity and ALDH**^**high**^**CD44**^**+ **^**cells within the ALDH**^**high **^**and ALDH**^**low **^**fractions of the three MPM cell lines. (A)** Representative FACS analysis of ALDH activity in H28, H2052 and Meso4 using Aldefluor assay. Baseline control of ALDH fluorescence was established by the addition of ALDH inhibitor, DEAB (+DEAB) and used to provide the ALDH^high^ region for cells without DEAB (- DEAB). The same gating principle was applied to sort for ALDH^high^ cells. **(B)** Representative FACS-based sorting of ALDH^high^ and ALDH^low^ fractions for H2052. The non-viable cells (PI^+^ cells) were excluded before gating the ALDH^high^ cells. In all cases, ALDH^low^ cells represent <4% of the dimmest cells relative to the analysed population. Average purity of ALDH^high^ cells was ≥98%. **(C)** The freshly-sorted ALDH^high^ and ALDH^low^ fractions were immediately re-stained with CD44 APC-H7 antibody to determine the co-expression of ALDH and CD44. The same procedures were employed for ALDH sorting of H28 and H2052 cell lines and to determine the co-expression of ALDH and CD44. Three independent experiments were done for each cell line.

We initially investigated for the presence of sphere-forming cells in the H28, H2052 and Meso4 MPM cell lines as tumour spheroids are believed to contain putative CSCs which may play a crucial role in chemoresistance [12,15,17-19]. Under anchorage-independent culture conditions, we found that all three MPM cell lines contained a cell population that generated spheres of different sizes (Figure 1A) with varying sphere-forming efficiencies (Table 1). On days 7–10, 1^st^ generation spheres were dissociated into single cells, resuspended in the sphere-forming media and observed for subsequent sphere generation. This was repeated until the 3^rd^ generation spheres were established. Our data showed that all of the MPM cell lines contained sphere-forming cells with the ability to produce spheres up to the 3^rd^ generation demonstrating self-renewal properties (Figure 1B). These findings provided evidence of the presence of putative CSCs which led us to identify these cells using the ALDH activity, a potential CSC marker in the lung believed to be partially responsible for resistance to cancer therapy [8,20]. Using the Aldefluor assay we measured varying percentages of ALDH^+^ cells in H28 (2.29% ± 1.6), H2052 (0.82 ± 0.32) and Meso4 (12.68 ± 7.3). Representative images of flow cytometry-based ALDH activity are shown in Figure 2A.

**Table 1:** Sphere-forming efficiency of the three MPM cell lines

**Cell line**	**Sphere generation**	**Sphere size (μm)**	**Sphere number/SD**	**Sphere efficiency (%)**
H28	1°	50 - 120	51/17.8	1.0
	2°	40 - 100	47.7/4.9	0.95
	3°	40 - 100	20/5.0	0.40
H2052	1°	100 - 600	11.5/5.7	0.23
	2°	120 - 500	20/6.2	0.40
	3°	100 - 500	25/7.4	0.50
Meso4	1°	80 - 600	14/5.7	0.28
	2°	40 – 500	12.5/5.3	0.25
	3°	40 - 500	10/5.0	0.20

Single-cell suspensions of H28, H2052 and Meso4 were seeded at 5 x 10^3^/well/ml onto a 24-well plate. Sphere-forming efficiency (%) was determined by dividing the number of spheres formed by the original seeded cells. The quotient was then multiplied by 100.

### ALDH^high^ and ALDH^low^ fractions show association with CD44

As has been described in a number of studies in solid tumours [4,8,18], we used the flow cytometry-based Aldefluor assay (Figure 2B) to select ALDH^high^ and ALDH^low^ subpopulations in order to demarcate a non-CSC from a CSC-like cell population in MPM cell lines, and determine the potential role of the former in chemoresistance. We also assessed whether the ALDH-sorted fractions co-express CD44 which would reveal a linkage between the two markers (Figure 2C). We found high percentages of ALDH cells co-expressing CD44 in the ALDH^high^ fractions of H28 (59.7%), H2052 (51.6%) and Meso4 (69.5%) relative to all tumour cells. The ALDH^low^ fractions also co-expressed CD44 although at much lower frequencies in H28 (1.8%), H2052 (2.0%) and Meso4 (1.1%) relative to all cancer cells. Taken together our data show the presence of ALDH^high^CD44^+^ subpopulations indicating an association of ALDH with CD44 within the ALDH^high^ and ALDH^low^ fractions of the three MPM cell lines.

### ALDH^high^ fractions show higher ALDH activity and generate robust, fast-growing sphere-forming cells

**Figure 3:**
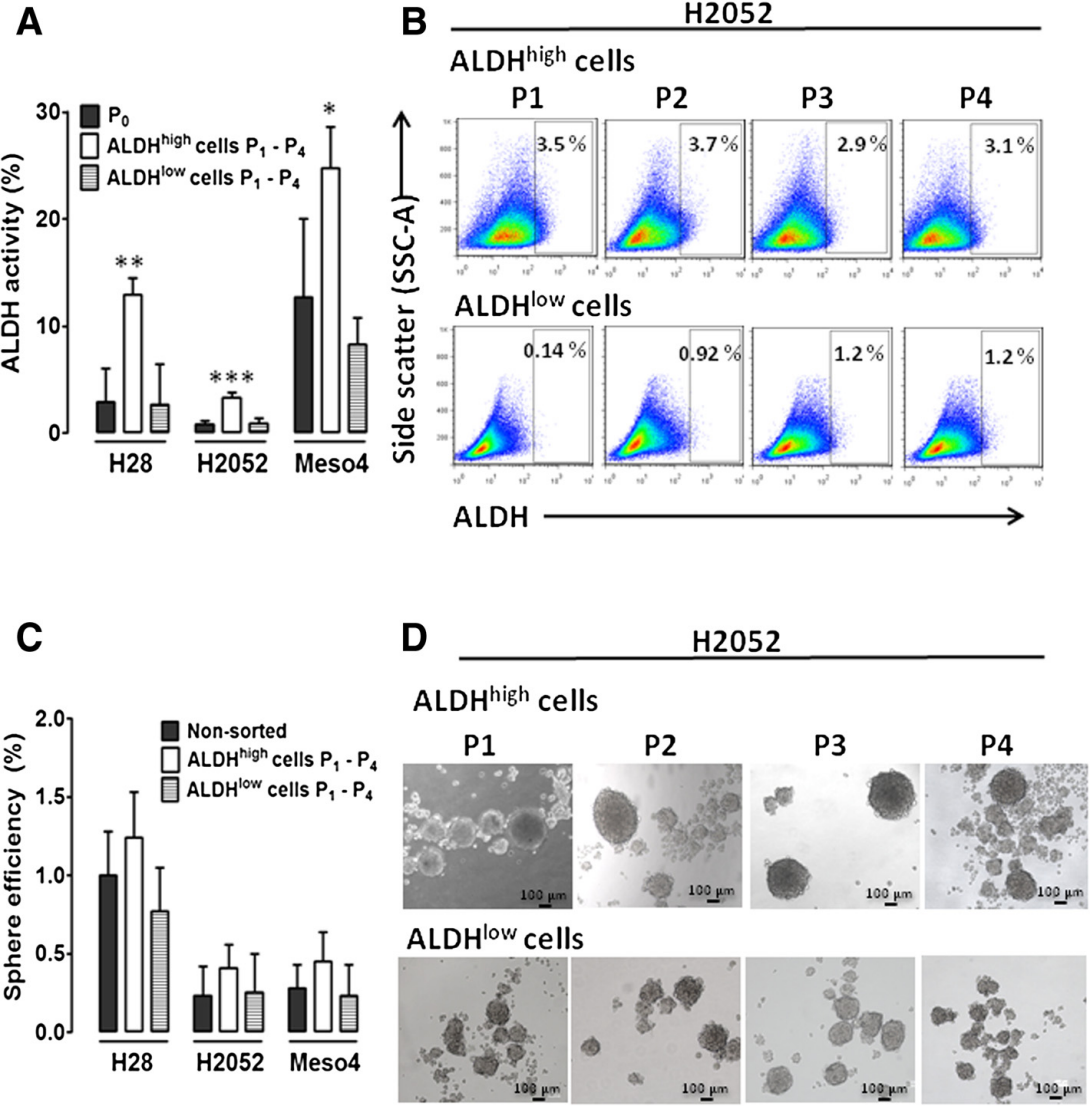
**Effect of ALDH sorting on ALDH activity and sphere-forming efficiency in H28, H2052 and Meso4 cell lines after in vitro expansion. (A)** The ALDH activity of the in vitro-expanded ALDH^high^ and ALDH^low^ fractions was determined by Aldefluor assay. P_0_ represents the ALDH activity of the non-sorted cell lines, whereas P_1_-P_4_ accounts for the average ALDH activity measured after 4 passages of in vitro expansion. **(B)** Representative FACS analyses of ALDH activity of ALDH-sorted fractions of H2052 measured at every passage. **(C)** The sphere-forming efficiency was also assessed after the in vitro expansion of the ALDH^high^ and ALDH^low^ fractions. Cells were grown under anchorage-independent cell culture conditions and were counted on day 7 for all cell lines and were used to determine the sphere-forming efficiency as described in the Methods. **(D)** Representative images of the sphere formation of ALDH-sorted fractions of H2052 taken at every passage. Results reflect the means and SDs of 3 independent experiments for each cell line. Results were statistically significant if *p* <0.05 (*p <0.05, **p <0.01).

We evaluated ALDH activity as well as the sphere-forming efficiency after the in vitro expansion of ALDH-sorted cells up to four passages. We determined ALDH activity at every passage and calculated the average increase from the four passages (P_1_-P_4_), which was compared with the non-sorted cells. The ALDH^high^ -sorted fractions showed a higher capacity to repopulate ALDH^+^ cells compared to the ALDH^low^ cells. Compared with the non-sorted cell lines (P_0_) we found significant 4.4, 4.0 and 1.9 - fold increases in the ALDH^high^-sorted H28, H2052 and Meso4 cell lines respectively. There were no significant differences between the ALDH^low^ cells and the non-sorted controls (Figure 3A). The ALDH^high^ cells and a much lower capacity of the ALDH^low^-sorted cells showed multilineage differentiation, and an ability to preserve ALDH activity up to the fourth passage. Representative images of flow cytometry-based ALDH activity analysed at every passage are shown in Figure 3B. We also tested the ability of ALDH^high^ and ALDH^low^ cells to produce spheres up to 4 generations under the anchorage-independent growth conditions. We observed a slight increase in the sphere-forming efficiency of ALDH^high^ fractions compared with the non-sorted cells. There were no differences in the ALDH^low^ fractions compared with the non-treated controls (Figure 3C). Notably, the spheres generated by the ALDH^high^ fractions in all of the cell lines grew faster, were larger and more robust under mechanical agitations compared with those derived from the ALDH^low^ cells and non-treated controls. Representative images of sphere formation taken at every passage are shown in Figure 3D.

### Cisplatin does not inhibit ALDH activity and sphere formation in both ALDH^high^ and ALDH^low^ fractions

**Figure 4:**
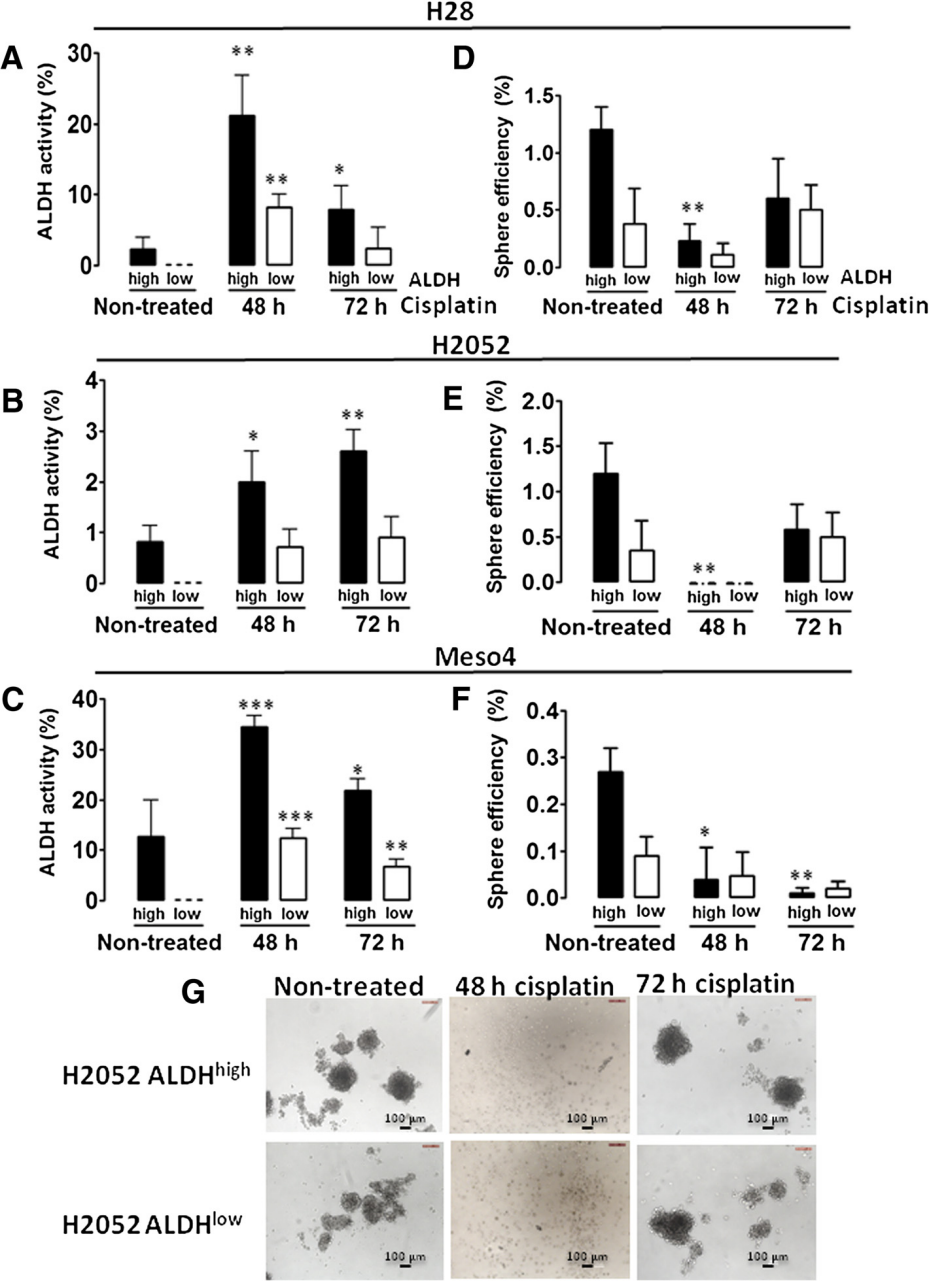
**Effect of cisplatin treatment on ALDH activity and sphere-forming efficiency.** Cells in 10 cm dishes were treated with the previously determined IC_50_ of cisplatin for ALDH-sorted fractions of the MPM cell lines. After the 48- and 72-h cisplatin treatments of ALDH^high^- and ALDH^low^-sorted cells, ALDH activity **(A-C)** was determined on surviving cells and compared with the non-treated cells to evaluate the effect of cisplatin by flow cytometry. The sphere-forming efficiency of the cisplatin-resistant cells was also evaluated as described in the Methods, and compared with the non-treated cells **(D-F)**. Results represent the means and SDs of 3 independent experiments each. Data are statistically significant if *p* <0.05 (*p <0.05, **p <0.01, ***p <0.001). Representative images of spheres from non-treated and cisplatin-treated H2052 ALDH^high^ - and H2052 ALDH^low^ -sorted cells **(G)**.

Considering the reported involvement of ALDH in the chemoresistance of different neoplasms [7,8,12,20,21], we assessed the drug response of ALDH-sorted fractions of the three MPM cell lines to the platinum-based chemotherapeutic drug, cisplatin, a standard drug in the treatment of MPM [1]. After 48- and 72-h treatments with the IC_50_ cisplatin: H28^high^ 28 μM, H28^low^ 8 μM; H2052^high^ 83 μM, H2052^low^ 27 μM; Meso4^high^ 27 μM, Meso4^low^ 9 μM (see Additional file 1), ALDH activity was determined by FACS analysis and compared with those of the non-treated cells. The relative enrichment as a result of drug treatment was taken as an indication of drug resistance of a cell population [22]. Despite the notable decrease in cell viability after cisplatin treatments in all of the ALDH-sorted cells of the three MPM cell lines (see Additional file 2), we found that 48-h drug treatment of the ALDH-sorted fractions in H28, H2052, and Meso4 showed greatly increased ALDH activity which decreased after 72-h drug exposure in H28 and Meso4 (Figure 4A, C). This effect was not observed in H2052 (Figure 4B). Our data show that the drug resistance of both ALDH^high^ and ALDH^low^ fractions of the MPM cell lines varies in response to cisplatin treatment, plausibly due to the differences in the pathological subtype given that both H28 and Meso4 are of a predominantly epitheloid phenotype and H2052 is sarcomatoid [23].

We further investigated the effect of this drug on the sphere-forming capacity of the ALDH-sorted fractions as the generation of spheres after drug treatment would reflect the presence of drug-tolerant CSCs [19]. After 48- and 72-h treatments with cisplatin, surviving cells were collected and incubated in a sphere-permissive medium. Sphere-forming efficiencies were compared with the non-treated cells. Figure 4D demonstrates that the 48-h drug treatment in H28 significantly attenuated sphere formation which increased after the 72-h drug exposure. In H2052, 100% elimination of the sphere-forming population was observed after 48 h of drug treatment, but unexpectedly re-appeared after 72 h (Figure 4E). Meso4 responded with a gradual reduction of the sphere formation with increased drug incubation time although this was not completely eliminated. (Figure 4F). Our data demonstrate that both ALDH^high^ and ALDH^low^ fractions of the MPM cell lines contain cisplatin-resistant sphere-forming cells. Representative images of sphere-forming cells of H2052 ALDH-sorted cells before and after cisplatin treatments are shown in Figure 4G.

### DEAB treatment prior to cisplatin exposure reduced cell viability but does not eliminate the sphere-forming cells in both ALDH^high^ and ALDH^low^ fractions

**Figure 5:**
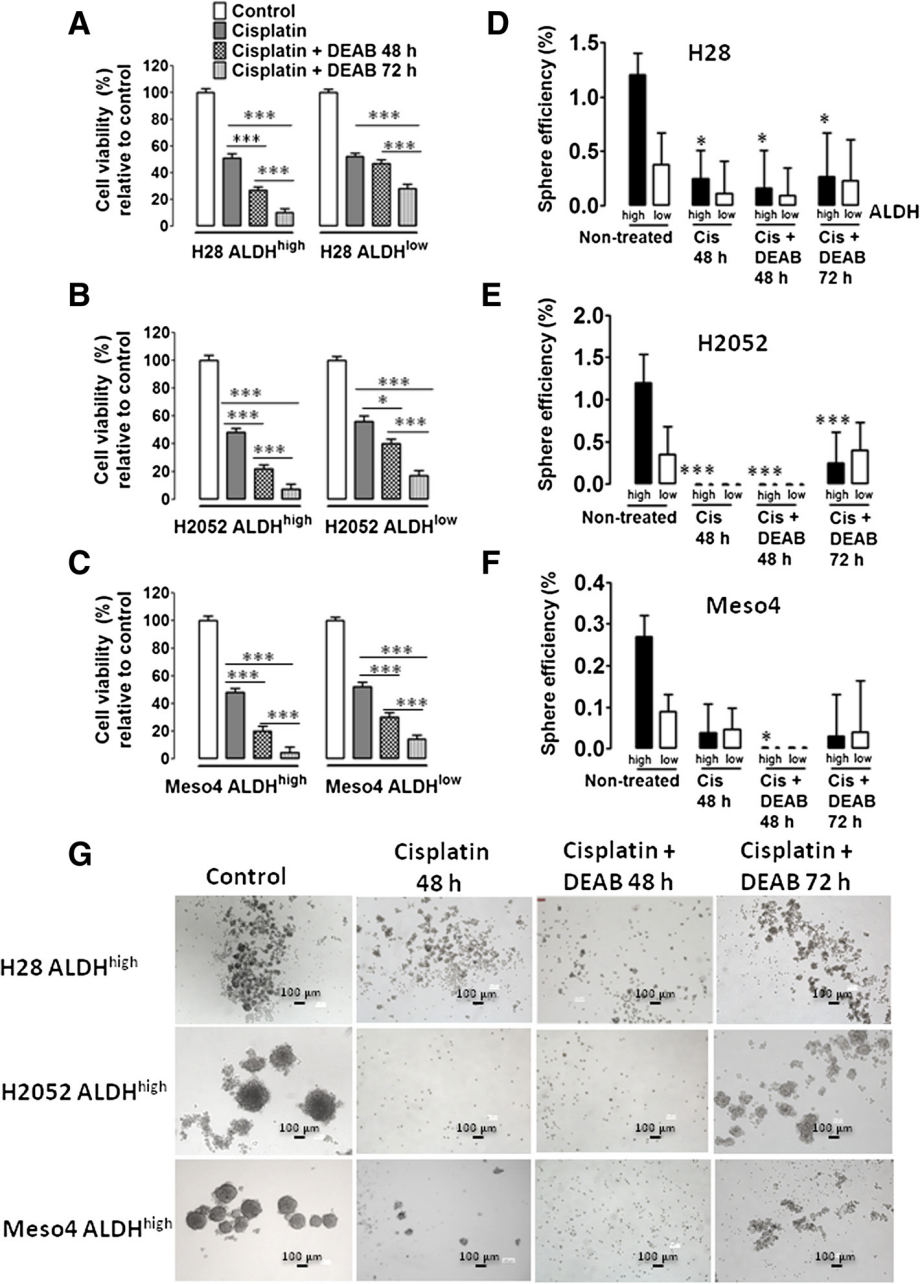
**Cisplatin + DEAB treatment decreases cell viability and induces a short-term inhibition of sphere formation.** ALDH^high^- and ALDH^low^-sorted cells of H28, H2052 and Meso4 were pre-treated with 100 μM DEAB for 48 h, then re-incubated with cisplatin (Cis) for either 48 h or 72 h. Cell viability of the surviving cells was quantified using tryphan blue exclusion test which was performed in triplicates in 3 independent experiments **(A-C)**. Surviving cells were allowed to form spheres under an anchorage-independent culture condition which were evaluated on day 7 and then compared with the non-treated cells **(D-F)**. Results represent means and SDs of 3 experiments with 6 replicates each. Dashed lines symbolize 0 values. The level of significance was set at *p* <0.05 (*p <0.05, **p <0.01, ***p <0.001). Representative images of sphere-formation of ALDH^high^ -sorted MPM cell lines, with and without cisplatin treatment, and in the presence or absence of 100 μM DEAB **(G)**. The ALDH^low^ -sorted fraction of each cell line showed the same effect (images not shown).

We hypothesized that specific inhibition of ALDH by DEAB may sensitize the ALDH-sorted fractions of the three MPM cell lines as DEAB has been shown to inhibit ALDH in breast, leukemia and lung cancer cell lines, as well as the ALDH^high^/CD44^+^ cells in breast cancer [6,14]. The sensitizing effect of DEAB was assessed by sequential 100 μM DEAB and cisplatin treatments and were evaluated by cell viability and sphere-forming efficiency. The 48-h cisplatin + DEAB treatments reduced cell viability in both ALDH^high^- and ALDH^low^-sorted fractions in all three MPM cell lines with remarkable reductions observed in the ALDH^high^ -sorted fractions of H28, H2052 and Meso4 compared with the cisplatin alone-treated cells. Prolonged treatment of 72 h markedly diminished the cell viability in all of the ALDH^high^ cells compared with the ALDH^low^ cells which demonstrated a less pronounced reduction in cell viability (Figure 5A-C). No decrease in sphere formation was found in H28 after 48 h of cisplatin + DEAB treatment compared with the cisplatin alone-treated cells (Figure 5D); H2052 maintained the absence of spheres, and the sphere-forming cells were totally eliminated in Meso4. Unexpectedly, and as previously shown in Figure 4D-F, 72-h incubation enhanced sphere efficiency in H28-sorted fractions, with the re-emergence of spheres in the ALDH^high^- and ALDH^low^-sorted H2052 and Meso4 cell lines (Figure 5D-F). These data indicate that DEAB exerts a marked reduction in cell viability of the ALDH^high^-sorted cells, but cannot fully sensitize the drug-resistant sphere-forming cells to cisplatin.

### ALDH isozymes and CD44 mRNA levels increased after cisplatin + DEAB treatment in both ALDH^high^ and ALDH^low^ sorted fractions

**Figure 6:**
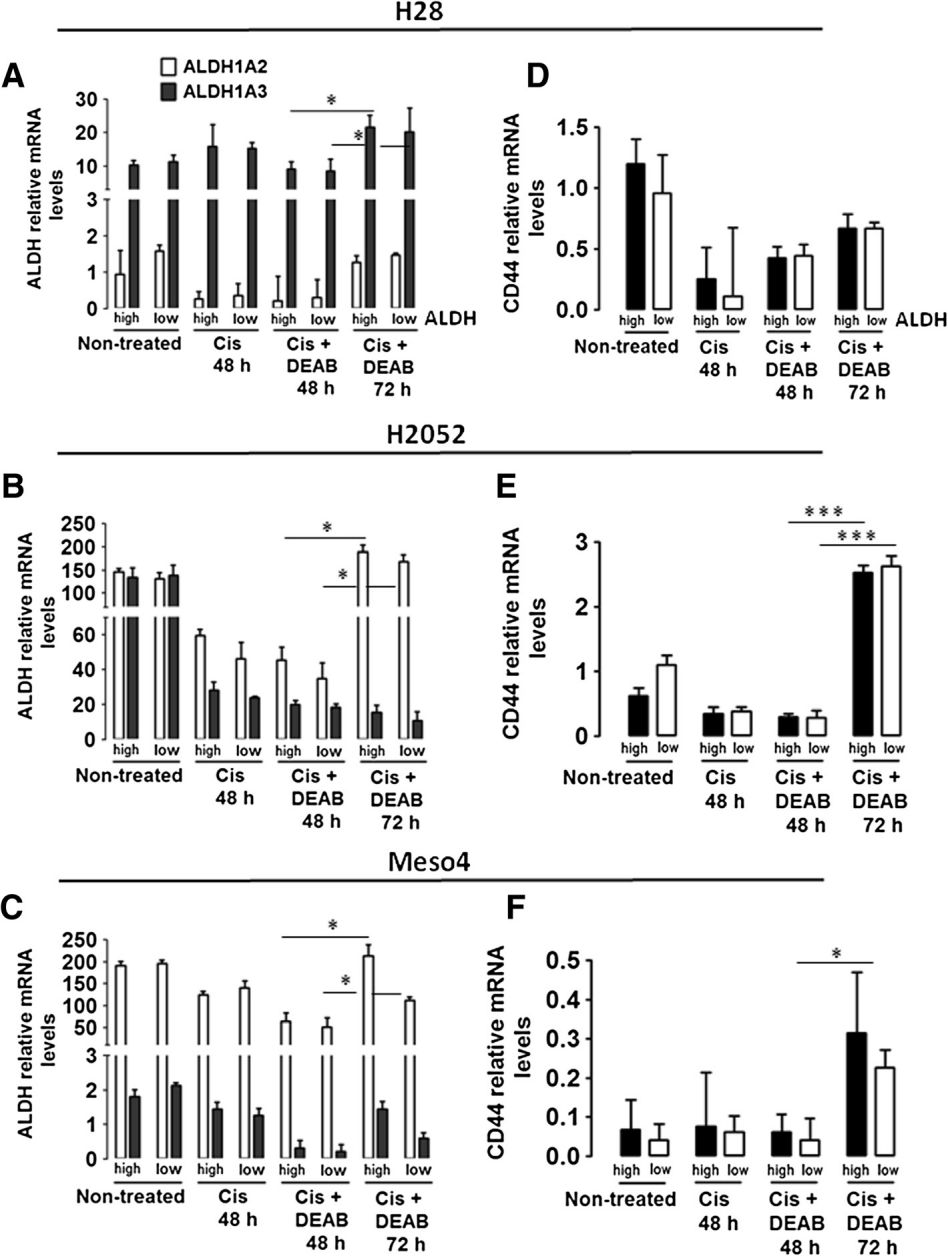
**Cisplatin + DEAB-resistant cells show increased mRNA levels of ALDH isozymes and CD44.** mRNA was isolated from non-treated and surviving cells of the ALDH-sorted fractions of H28, H2052 and Meso4 after cisplatin treatment in the presence or absence of DEAB for 48 and 72 h. Relative mRNA expression levels of ALDH1A2 and ALDH1A3 **(A-C)** and CD44 **(D-F)** were determined by qRT-PCR using the ΔΔCT method. Histograms represent the means and SDs from 3 independent experiments each. Results are statistically significant if *p* <0.05 (*p <0.05, ***p <0.001).

The failure of cisplatin + DEAB treatment to inhibit sphere formation led us to examine the drug-resistance properties of ALDH and CD44 at the transcript level. We analysed the mRNA levels of the two markers in the non-treated, cisplatin-treated and cisplatin + DEAB-treated cells in the ALDH-sorted fractions of the three MPM cell lines. Aldefluor assay can specifically detect ALDH1A, ALDH1A2, ALDH1A3 and ALDH2 [6,24]. We therefore evaluated the four ALDH isozymes in the surviving cells after the drug treatments using qRT-PCR analysis. Increased mRNA levels after the 72-h cisplatin + DEAB treatment reflects drug tolerance as demonstrated by the re-ermergence of sphere formation. ALDH1A2 and ALDH1A3 are the predominant isozymes in the three MPM cell lines. In H28, cisplatin + DEAB treatment reduced ALDH1A3 after 48 h but this was significantly increased after 72 h. ALDH1A2 was also upregulated but not significantly (Figure 6A). In H2052 and Meso4, cisplatin + DEAB incubations for 48 h slightly reduced ALDH1A2 and ALDH1A3, but remarkably, only ALDH1A2 was significantly enhanced after the 72-h treatment (Figure 6B-C). As expected, cisplatin + DEAB treatment for 48 h did not confer an inhibitory effect on CD44 in the ALDH-sorted fractions of H2052 and Meso4, but slightly increased it in H28. Importantly, CD44 significantly increased in both ALDH- sorted fractions of H2052 and the Meso4ALDH^high^ fraction (Figure 6E-F). Meso4ALDH^low^ fraction and H28-sorted fractions also showed higher mRNA levels but did not reach significance (Figure 6D, F). These results highly indicate that ALDH 1A2, ALDH1A3 and CD44 confer resistance to cisplatin + DEAB; hence, the cells bearing these markers may partially account for the re-appearance of spheres after the drug treatment.

## Discussion

The identification of a chemoresistant population offers the possibility of cell-specific therapeutic approaches that may augment the current treatment modalities in malignant pleural mesothelioma. Here we show that ALDH^high^CD44^+^ cells are involved in conferring resistance to cisplatin in the three MPM cell lines; H28, H2052 and Meso4. We also demonstrate that ALDH as a single marker is not sufficient to define chemoresistant, sphere-forming cell populations in the tested MPM cell lines.

Several reports have supported the utility of ALDH to identify CSC-like populations in different malignancies and its potential as a therapeutic target [5,8,20,25,26]. We found that all MPM cell lines are capable of producing spheres for three consecutive generations thus sustaining the property of self-renewal, a stem cell feature considered as a key discriminating difference between CSCs and non-CSCs [27]. The presence of ALDH activity in H28, H2052 and Meso4 further supports the existence of putative CSC populations in these cell lines. Unexpectedly, the ALDH^high^- and ALDH^low^-sorted cells which were supposed to demarcate a CSC- from non-CSC-like cells showed sphere-forming ability and generate ALDH activity although the ALDH^low^ cells were less efficient. They also both showed an association with CD44. Wang et al. [12] and Prasmickaite et al. [22] also found that ALDH^br/+a^nd ALDH^low/-^ cells repopulated stem cell heterogeneity, formed spheroids and generated tumours in epithelial and malignant melanoma. In the lung adenocarcinoma cell line, SPC-A1 both ALDH^lo^ and ALDH^high^-sorted cells formed colonies and developed tumours although ALDH^lo^ was less efficient [28]. In the MPM cell line, MSTO211H and Ewing’s sarcoma cell lines only purified ALDH^bright^ cells generated both ALDH^bright^/ALDH^low^ cells, but ALDH^low^ did not repopulate ALDH^bright^ cells [21,29]. These studies suggest that the type of tumour and heterogeneity within a cancer [30] are at least in part, responsible for the differential behavior of ALDH in the malignant setting. Moreover, ALDH activity may be dependent on whether the tumour conforms to the cancer stem cell model. The CSC model proposes the presence of a cellular hierarchy in the tumour, and that only a subset of tumour cells possess the ability of self-renewal and to generate the different phenotypes that comprise the neoplasm [31]. A conversion of ALDH^low^ cells into ALDH^high^ cells in the presence of an appropriate environment during the short-term, in vitro culture is a possible explanation. This assumption follows the stemness phenotype model (SPM) proposing that all cancer cells possess stem cell properties, and that stemness is modulated by the environment such that CSCs and non-CSCs can interconvert into each other when changes in the environment favours this conversion [32]. In support of this hypothesis, it has been shown that CD44^-^ Du145 prostate cells produced CD44^+^ cells in vitro [33], and in the MCF-7 breast cancer cell line, the sorted non-SP cells gave rise to SP cells [34]. The question of whether MPM follows the CSC or SMP model warrants further investigation.

Our findings showed that ALDH activity is resistant to cisplatin treatment in the ALDH-sorted fractions of the three MPM cell lines. This consolidates with the biological function of ALDH in its ability to detoxify anticancer drugs such as oxazaphosphorines, cyclophosphamides and taxanes thus conferring drug resistance [5,7,14]. Consistent with our findings, chemoresistance as an attribute of ALDH^+^ cells has been documented in different solid tumours including lung cancer and primary MPM specimens [8,21,26,29,35]. The presence of ALDH^high^CD44^+^ subpopulations in the ALDH-sorted fractions highly indicates that in addition to ALDH, CD44 may also contribute to cisplatin resistance. The current consensus posits that CD44^+^ subfractions in many human cancers are highly malignant and drug resistant. In non-small cell lung cancer, CD44^+^-sorted cells with stem cell-like properties were found to be more resistant to cisplatin than CD44^-^ cells [15]. Wang et al. [12] proposed that the combination of ALDH1 and CD44 stringently defined ovarian cancer stem cells, which showed chemoresistance and poor clinical clinical outcome. This is strengthened by the recent evidence that ALDH^hi^CD44^hi^ tumour-initiating cells maintained lung tumorigenicity and drug resistance in patient-derived lung cancer cells [36].

Our results demonstrated that sequential DEAB and cisplatin treatments remarkably diminished cell viability in the ALDH^high^-sorted cells, a short-term elimination of spheres, but not a complete inhibition of sphere formation in all of the ALDH^high^ and ALDH^low^ fractions of MPM cell lines. The profound effect of DEAB in the cell viability of ALDH^high^ fractions is supported by the findings which showed that the downregulation of ALDH isozymes in A549 lung cancer cell line altered cell proliferation and motility, whereas an analogous experiment in cell lines devoid of ALDH-expressing cells had an insignificantly less inhibitory effect on cell proliferation demonstrating a functional role of ALDH in the regulation of cell growth [9]. Croker and Allan [14] also observed a significant reduction in cell viability but not a complete inhibition of a long-term re-growth of colony-forming ability of chemo- or radiation-treated ALDH^hi^CD44^+^ cells pre-treated with DEAB in breast cancer. A plausible explanation for the re-emergence of spheres may be attributed to the survival of putative CSCs which escaped the cytotoxic effect of cisplatin, and were tolerant to DEAB treatment. We speculate that the tested MPM cell lines have a heterogeneous cancer cell population. The more differentiated cells within the hierarchy might be efficiently killed by chemotherapy, whereas the less differentiated cells bearing CSC phenotypes survive and give rise to new transit-amplifying cells with the capacity to regenerate the culture [37]. The possibility of cells existing in a dormant quiescent state with the capacity to regrow when the environmental cues are appropriate could also be an attribute [38]. If the observed cisplatin resistance is unique to ALDH^high^ cells only, then a DEAB-mediated sensitization process should have prevented the re-growth of spheres. The failure of DEAB to sensitize both the ALDH-sorted fractions to cisplatin strongly supports our assumption that the ALDH^high^CD44^+^ cells are crucial players in conveying drug tolerance. Hence, specific targeting of both phenotypes may offer a more effective chemotherapy.

Studies have shown that ALDH activity may reflect other ALDH isozymes in addition to the prevalent ALDH1A1 which are important in the regulation of several biological activities including drug resistance [39,40]. Hence, the identification of specific isozymes contributing to ALDH activity is a critical factor. We observed an upregulation of ALDH1A2 in H28 and ALDH1A3 in H2052 and Meso4 in the cisplatin + DEAB-resistant cells of the ALDH-sorted fractions of the three MPM cell lines, indicating the implication of these two isozymes in conveying resistance to cisplatin and DEAB. Other groups [6,8,28] have detected ALDH1A1, ALDH1A2, ALDH3A1, and ALDH2A1 in lung cancers which were shown to have an association with chemoresistance. CD44 was likewise increased after an analogous treatment with cisplatin + DEAB highly suggestive of an essential role in conferring drug tolerance. Our data indicate that at the transcript level, ALDH and CD44 are important players in the observed resistance to cisplatin and DEAB. Notably, inhibition of ALDH activity cannot sensitize the ALDH^hgh^CD44^+^ cells to drug treatment.

## Conclusions

In conclusion, we found that both ALDH^high^ and ALDH^low^ fractions of the three MPM cell lines; H28, H2952 and Meso4 harboured ALDH^high^CD44^+^ cells that are involved in conferring resistance to cisplatin, and may therefore serve as potential therapeutic targets to improve the current treatment modalities in MPM. Our data also demonstrate that the double expression of ALDH and CD44 rather than ALDH alone better delineates a chemoresistant, sphere-forming cell populations in the tested MPM cell lines. Further investigations are necessary to determine the distinct role of ALDH^high^CD44^+^ cells in fueling chemoresistance to other anti-neoplastic drugs in the treatment of MPM.

## Abbreviations

CSCs: Cancer stem cells; ALDH: Aldehyde dehydrogenase; IC50: Inhibitory concentration 50; SDs: Standard deviations; EGF: Epidermal growth factor; bFGF: basic fibroblast growth factor; FACS: Fluorescence activated cell sorting; XTT: Tetrazolium salt used in XTT cell viability assay.

## Competing interests

The authors declare that they have no conflict of interest.

## Authors’ contribution

LCD conceived and designed the study, analyzed the data and wrote the manuscript. LF and RB performed cellular and RT-qPCR assays, flow cytometry-based analyses, collected and graphed experimental data. RAS gave suggestions to improve the impact of the study and approved the final version of the manuscript for publication. GK made significant contributions to the design of the study, analysis of data and critically improved the manuscript for intellectual content. All authors read and approved the final manuscript.

## Pre-publication history

The pre-publication history for this paper can be accessed here:

http://www.biomedcentral.com/1471-2407/14/304/prepub

## Supplementary Material

Additional file 1 **Dose–response curves of MPM cell lines to cisplatin.** Effect of the different concentrations of cisplatin (0 – 256 μM) on the cell viability of ALDH^high^-sorted cells (red), ALDH^low^-sorted cells (green) and non-sorted cells (blue) of H28 **(A)**, H2052 **(B)**, and Meso4 **(C)** as determined by the XTT assay. Results represent the means and SDs of 3 independent experiments. Click here for file

Additional file 2 **Effect of cisplatin treatment on cell viability.** Cells grown in 10 cm dishes were treated with the corresponding IC_50_ of cisplatin for ALD^high^- and ALDH^low^- sorted cells of the three MPM cell lines. After 48- and 72-h incubations, XTT assay was performed to determine the number of viable cells relative to control. Results represent the means and SDs of three independent experiments. Data are statistically significant if p <0.05 (*p <0.05, **p <0.01, ***p <0.001). Click here for file
