# Supplementary figures and images for: Cisplatin-resistant cells in malignant pleural mesothelioma cell lines show ALDHhighCD44+ phenotype and sphere-forming capacity
Source: BMC Cancer. 2014 Apr 30;14:304. doi: 10.1186/1471-2407-14-304 (PMC4021184; doi:10.1186/1471-2407-14-304)

**A****H28**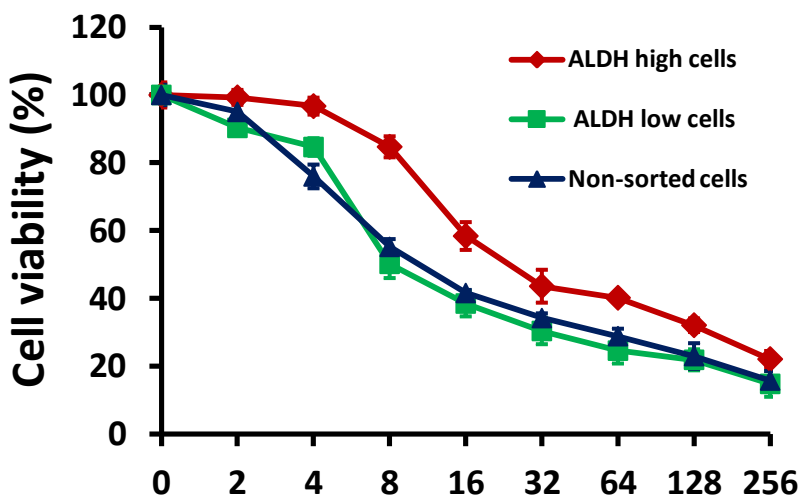**B****H2052**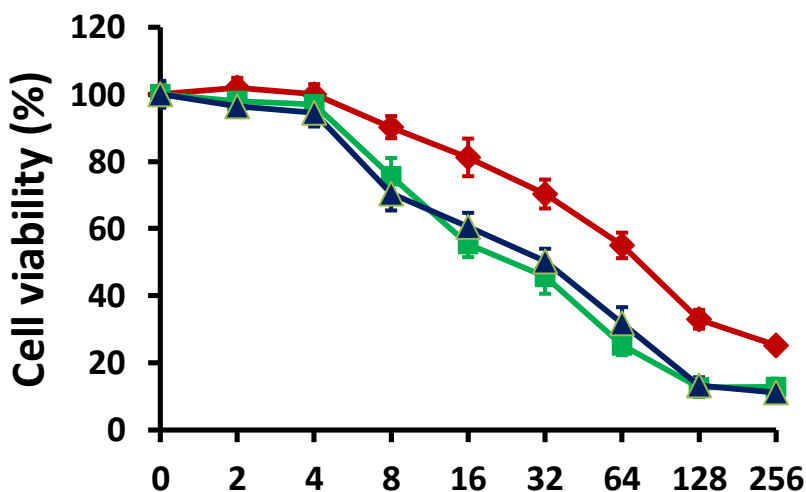**C****Meso4**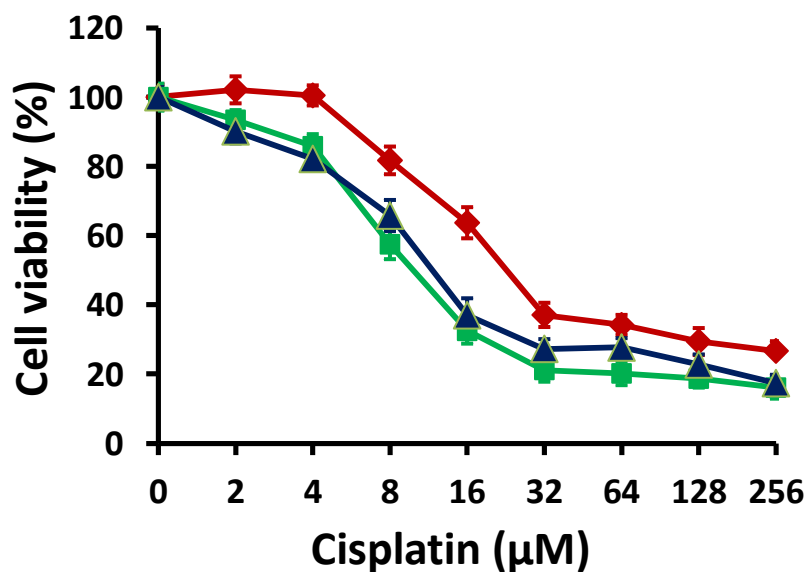

Supplement: Additional file 1 — Dose–response curves of MPM cell lines to cisplatin. Effect of the different concentrations of cisplatin (0 – 256 μM) on the cell viability of ALDHhigh-sorted cells (red), ALDHlow-sorted cells (green) and non-sorted cells (blue) of H28 (A), H2052 (B), and Meso4 (C) as determined by the XTT assay. Results represent the means and SDs of 3 independent experiments. [file 1471-2407-14-304-S1.pdf]

**A****H28**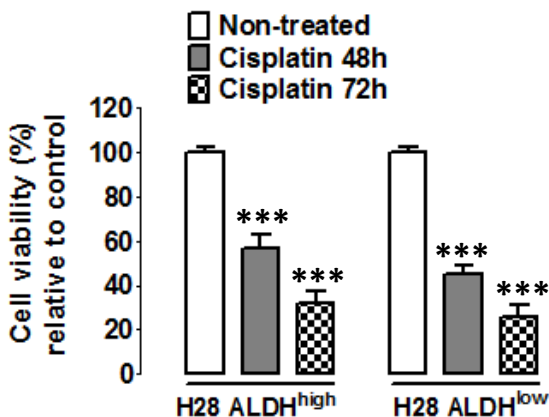**B****H2052**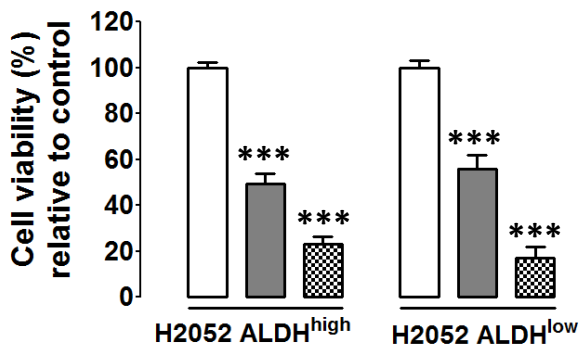**C****Meso4**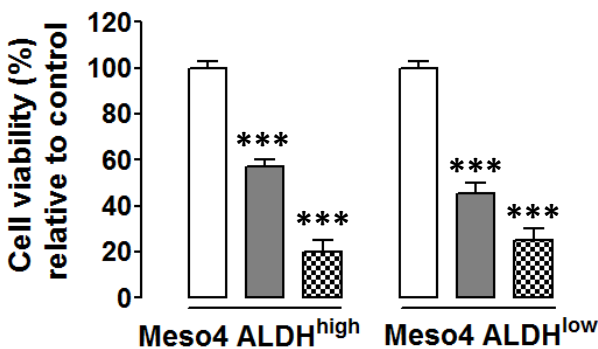

Supplement: Additional file 2 — Effect of cisplatin treatment on cell viability. Cells grown in 10 cm dishes were treated with the corresponding IC50 of cisplatin for ALDhigh- and ALDHlow- sorted cells of the three MPM cell lines. After 48- and 72-h incubations, XTT assay was performed to determine the number of viable cells relative to control. Results represent the means and SDs of three independent experiments. Data are statistically significant if p <0.05 (*p <0.05, **p <0.01, ***p <0.001). [file 1471-2407-14-304-S2.pdf]
